# Expression of lamina proteins Lamin and Kugelkern suppresses stem cell proliferation

**DOI:** 10.1080/19491034.2017.1412028

**Published:** 2018-01-23

**Authors:** Roman Petrovsky, Jörg Großhans

**Affiliations:** Institute of Developmental Biochemistry, University Medical Center, University of Göttingen, Justus-von-Liebig Weg 11, 37077 Göttingen, Germany

**Keywords:** Drosophila, Jak/Stat, Kugelkern, Lamin, nuclear lamina, stem cell

## Abstract

The nuclear lamina is involved in numerous cellular functions, such as gene expression, nuclear organization, nuclear stability, and cell proliferation. The mechanism underlying the involvement of lamina is often not clear, especially in physiological or developmental contexts. Here we investigate the role and activity of farnesylated lamina proteins Lamin (Lam) and Kugelkern (Kuk) in proliferation control of intestinal stem cells (ISCs) in adult *Drosophila* flies. We found that ISCs mutant for *Lam* or *kuk* proliferate, whereas overexpression of Lam or Kuk strongly suppressed proliferation. The anti-proliferative activity is, at least in part, due to suppression of Jak/Stat but not Delta/Notch signaling. Lam expression suppresses Jak/Stat signaling by normalization of about 50% of the Stat target genes in ISCs.

## Introduction

Overexpression of Lamins including prelamin A and the progeric Lamin A variant Progerin promote nuclear deformations, DNA damage, reduced heterochromatin and reduced lifespan [1,2,3]. The *Drosophila* genome contains a single B-type Lamin Dm (Lam) and a single A-type lamin the non-farnesylated Lamin C (LamC) [4,5,6]. In addition, *Drosophila* contains the farnesylated lamina protein Kugelkern (Kuk) [7] Although structurally distinct from lamins, Kuk shares several functional elements with lamins. A C-terminal farnesylation motif, a putative coiled coil motif and a nuclear localization signal (NLS) [7]. Kuk is functionally related to lamins, since expression of Kuk induces similar phenotypes in several cellular systems [7,8,9], including abnormal nuclear morphology, decreased heterochromatin and increased DNA damage [7,8,9]. The function and activity of lamina proteins on proliferation control and tissue homeostasis is not clear. Specifically in stem cells, Prelamin A and Progerin have been shown to change proliferation and homeostasis [10,11]. Adult *Drosophila* flies contain three populations of proliferative stem cells: germ cells, somatic follicle stem cells and intestinal stem cells in the midgut epithelium (ISC)[12,13,14,15,16]. Here we aim to investigate the role and activity of Lam and Kuk within the midgut of adult *Drosophila* flies.

**Figure 1.**
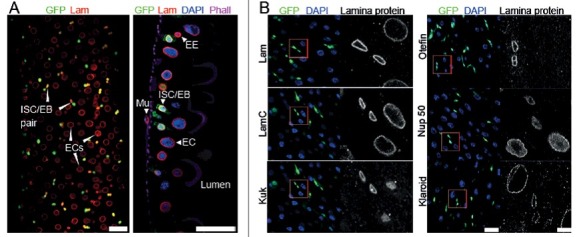
Expression of lamina proteins in the midgut. (A) Fixed adult midguts stained for GFP, Lam, DNA and F-actin. Color coding as indicated. Surface view and sagittal section. Intestinal stem cells (ISC) and enteroblast (EB) were genetically labelled by esg-GAL4 driven expression of GFP (green). Enterocytes (EC), enteroendocrine cell (EE), viceral muscle cell (Mu). Scale bars 25 μm. (B) Fixed midguts stained for indicated proteins of the nuclear envelope (gray), ISC/EBs (green), DNA (blue). Asterics: Nucleoplasmic Kuk staining in EC is unspecific as it is also observed in *kuk* deficient flies. (Fig. S1). Scale bars 25 μm, 5 *µ*m, inset in 4x magnification.

The midgut has a simple morphological organization containing five major cell types: intestinal stem cells (ISC) [17,18], enteroblasts (EB), absorbtive enterocytes (EC), secretory enteroendocrine cells (EE), and visceral muscle cells (Fig. 1A). These five cell types can be genetically and histologically distinguished [15,16,19,20] Additional cell types are found in the copper cell region of the midgut, which we do not consider in this study. Under laboratory conditions, the midgut tissue in females turns over within about two weeks. The levels of proliferation is low with a few mitotic events per gut [21]. The midgut displays a high capacity of regeneration. It quickly responds to infection or damage by a strong ISC proliferation with an up to 10x higher mitotic index and rapid tissue turnover [18].

ISC proliferation is regulated by a set of signaling pathways. ECs and EBs promote ISC division by secretion of cytokines or mitogens, which induce Jak/Stat, EGFR/Ras/MAPK, Hippo and Wg/Wnt signaling in the ISCs [21,22,23,24,25]. Visceral muscle cells induce canonical Wnt, Jak/Stat and EGFR signaling to promote stem cell maintenance over a longer time span and insulin signaling in response to food uptake [19,26,27,28,29]. ISCs and enteroblasts often stay in close proximity and regulate their identity and proliferation by Delta/Notch signaling [15,16,20].

Here we investigate whether and how farnesylated proteins Lam and Kuk influence ISC proliferation in adult *Drosophila*. We found that overexpression of both Lam and Kuk strongly suppresses ISC proliferation during homeostasis and regeneration. Lam antagonizes the regulation of the cell cycle by suppressing Jak/Stat signaling on the level of transcription, as indicated by normalization of half of the Jak/Stat target genes in ISCs.

## Results

We first analyzed the dynamics of lamina proteins in the midgut of adult *Drosophila* [30,31,32]. Generally, B-type lamins are ubiquitously expressed, whereas A-type lamins are assumed to show cell type specific expression [10]. We stained fixed midguts with a panel of antibodies, specific for lamina proteins, to establish their dynamics during differentiation. ISC/EBs were marked by GFP, enterocytes recognized by cell size and polyploid nuclei. We found that Kuk and the LEM domain protein Otefin specifically marked ISC/EBs. Lam showed stronger staining in ISC/EBs than in ECs, whereas Lamin C (LamC) and the SUN domain protein Klaroid stained uniformly in both ISC/EBs and ECs. The staining for the nuclear pore protein Nup50 was slightly stronger in ECs as compared to ISC/EBs (Fig. 1B, S1).

### Lam and Kuk inhibit ISC proliferation

**Figure 2.**
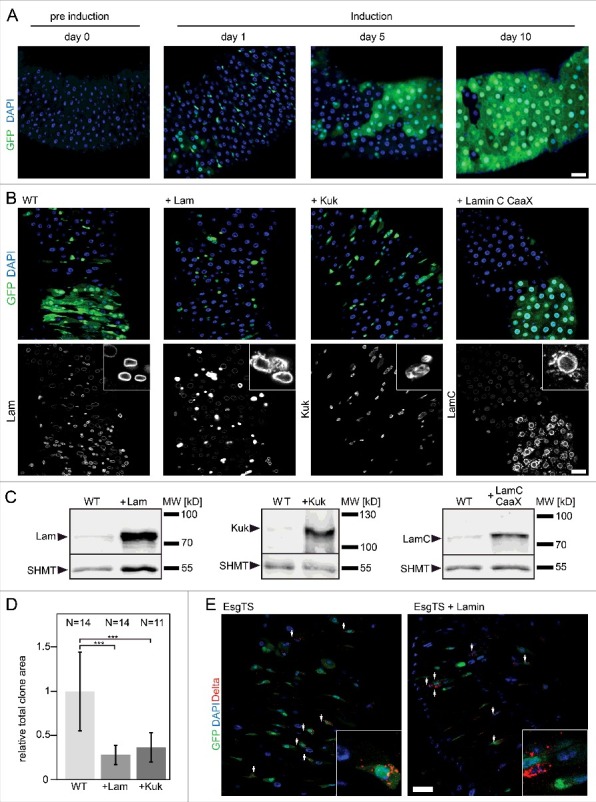
*Lam* overexpression inhibits ISC proliferation. (A, B) Flipout clones (green) in fixed adult midguts expressing GFP (A) or GFP and *Lam* or *kuk* or *LamC-CaaX* (B) stained for DNA (blue), GFP (green) and indicated lamina protein (grey). (A) Period after clone induction is indicated. (B) Five days after clone induction. The size of clones reflects proliferation during the period after clone induction. Inset, 3x magnification. (C) Expression levels of Lam, Kuk and LamC-CaaX analyzed by western blots with total gut extracts. SHMT serves as loading control. (D) Quantification of clone size expressing GFP (WT) Lam (+Lam) or Kugelkern (+Kuk). The area with GFP expression marking the clone(s) in relation to total gut area within the field of view. Five days after clone induction. Bars represent standard deviation. N, number of guts. Statistical significance was tested by students T-test, two tailed, two-sample unequal variance. P(WT vs. +Lam) = 3.6×10^−5^, P(WT vs. +Kuk) = 1.3×10^−4^. (E) Fixed midguts expressing GFP in ISC/EBs driven by Esg-GAL4 stained for GFP (green), DNA (blue) and the stem cell marker Delta (red). Expression was induced for 15 d. Arrowheads point to Delta and GFP positive cells. Insets 4x magnification. Scale bar 25 μm.

We reported previously that overexpression of Lam and Kuk in adult tissue such as muscle or fat body induces changes in nuclear morphology [8]. Here, we overexpressed Lam and Kuk in ISC/EBs and their progeny. For this, we employed a driver line (Esg-GAL4), which specifically expresses UAS transgenes in ISC/EBs. Expression in the progeny was induced by a flipout cassette. Expression and flipout was induced by a shift in temperature. Please see methods for experimental details [21]. As a measure for proliferation, we determined the relative size of marked clones. Consistent with previous work [21], the size of clones gradually increased until a full turnover was reached after about two weeks (Fig. 2A, S2). In contrast, clonal expansion was strongly inhibited by overexpression of Lam and Kuk. Lam overexpressing clones largely consisted of individual cells even after ten days. In the case of Kuk, small clones formed, consisting of a few cells. Immunostaining and western blots confirmed effective overexpression of Lam or Kuk within the clones (Fig. 2B, C, D and S3). The phenotype is specific for Lam and Kuk, as expression of a farnesylated variant of LamC (LamC-CaaX) did not affect clonal expansion (Fig. 2B). The suppression of proliferation by overexpression of Lam might be due to a loss of the stem cell character. To test this option, we overexpressed Lam exclusively in ISC/EBs and stained for Delta, which labels stem cells. Even after 15 days, we detected Delta staining in the small cells overexpressing Lam (Fig. 2E). These data indicate that the stem cell identity was maintained even after long term Lam overexpression.

**Figure 3.**
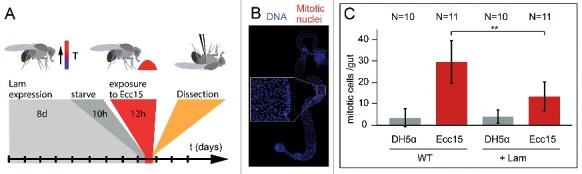
*Lam* overexpression inhibits pathogene induced proliferation. (A) Experimental scheme for *Lam* overexpression and *Ecc15* infection. (B) Fixed gut stained for the mitotic marker pH3 (phospho-S10-Histone 3, red) and DNA (blue). Inset, higher magnification showing mitotic nuclei. (C) Mitotic cells per gut. Guts from flies expressing GFP or GFP and *Lam* and infected with *E. coli* (DH5α) or pathogenic *Ecc15* were stained for the mitotic marker pH3. Bars represent standard deviation. N, sample size. Statistical significance was tested by Students T-test, two tailed, two-sample unequal variance. P(WT vs. *Lam*) = 9.2×10^−4^.

In addition to homeostatic conditions, we also tested proliferation during regeneration of the gut after bacterial infection. Feeding flies with pathogenic bacteria, such as *Ecc15*, induces a boost in ISC proliferation [18]. Following overexpression of Lam in ISC/EBs for eight days and short starvation, flies were fed with *Ecc15* bacteria (Fig. 3A). We measured the proliferative response by the mitotic index (Fig. 3B). Compared to control flies, which were fed by non-pathogenic laboratory stain, *E. coli* (DH5α), infection by *Ecc15* bacteria significantly increased proliferation. Comparable to homeostatic proliferation, overexpression of Lam reduced infection-induced proliferation to about half (Fig. 3C).

In summary, we found that overexpression of Lam or Kuk suppresses ISC proliferation under homeostatic as well as regenerative conditions. The induced inhibition of proliferation is specific as the LamC variant LamC-CaaX was not effective.

### Lam overexpression does not inhibit Notch/Delta induced proliferation

**Figure 4.**
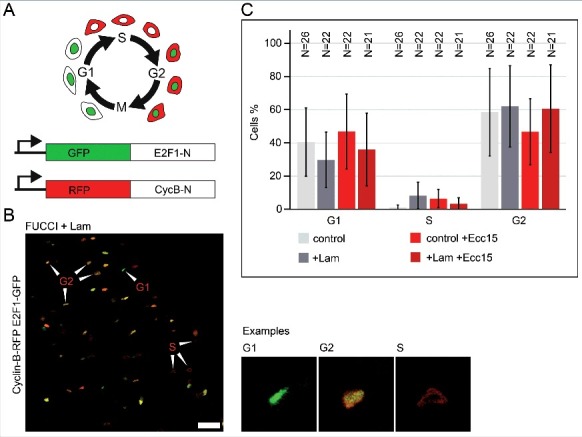
*Lam* overexpression does not change the distribution of the cell cycle stages. (A) Schematic of the FUCCI system. Labelling of G1, S and G2 phase by two reporter proteins (GFP-E2F1-N or RFP-Cyclin B-N). (B) Fixed midgut expressing FUCCI reporters in ISC/EBs. Arrowheads point to exemplary cells for G1, G2 or S phase. Examples shown in high magnification. Scale bar 25 μm. (C) Quantification of cell cycle stage distribution. Genotypes and infection as indicated. *Lam* was overexpressed in ISC/EBs with Esg-GAL4TS. N, sample size. Bars represent standard deviation.

The activity of Lam may be due to specific modulation of the cell cycle, e.g. activation of a checkpoint and cell cycle stage-specific arrest. Alternatively, Lam may act indirectly by suppression of a proliferative signal. We scored the distribution of cell cycle stages, G1, S, G2 in the ISC/EB cells, employing the so-called FUCCI system [33] (Fig. 4A and B). A high proportion of a specific stage would indicate a specific arrest of the cell cycle. In control guts, about two thirds of ISC/EBs resided in G2 phase and one third in G1 phase. The proportion of ISC/EBs in S phase was low, which is consistent with the low proliferation rate during homeostasis (Fig. 4C). Following *Ecc15* infection, the proportions of G1, G2 and S-phase were not obviously changed. Overexpression of Lam in ISC/EBs also had no obvious influence on stage distribution under homeostatic conditions and following *Ecc15* infection. We observed a wide experimental variation, which may obscure potentially subtle effects. With this limitation we conclude that Lam overexpression does not induce a specific checkpoint or cell cycle arrest.

**Figure 5.**
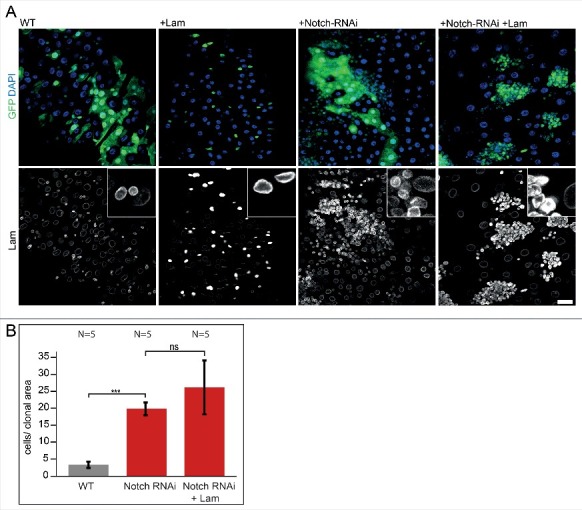
*Lam* overexpression does not inhibit *Notch/Delta* induced proliferation. (A) Fixed midguts with clonal expression of GFP (WT) or GFP and *Lam* and *Notch* RNAi as indicated (green, GFP) and stained for Lam (grey) and DNA (blue). Five days of induction time. Scale bar 25 μm. Insets, 4x magnification.(B) Quantification of clone size (cells per clonal area (μm^2^/1000). The area with GFP expression was related to the total gut area within the field of view. Five days after clone induction. Bars represent standard deviation. N, number of gut regions analyzed. Statistical significance was tested by students T-test, two tailed, two-sample unequal variance. P(WT vs. *Notch* RNAi) = 6.9×10^−8^, P(*Notch* RNAi vs. *Notch* RNAi +*Lam*) = 8×10^−2^.

To provide support for a specific regulatory function of Lam, we sought for a proliferative situation independent of Lam. Activation of the Notch/Delta pathway is a potent inducer of proliferation. Consistent with previous reports [20], we observed a tumor-like proliferation after expression of *Notch* dsRNA, as characterized by the size of clones and high number of small cells per clonal area (Fig. 5A, B and S4). This strong proliferation was not suppressed by coexpression of *Lam* and *Notch* dsRNA. Immuno-staining for Lam confirmed induced overexpression of Lam in clones (Fig. 5 A). We conclude that Lam overexpression is compatible with cell cycle progression as it does not inhibit the cell cycle in all circumstances. The data suggest that Lam does not directly target the cell cycle machinery but rather an upstream regulatory signal independent of Notch/Delta.

### Lam and Kuk overexpression inhibits Jak/Stat induced proliferation

**Figure 6.**
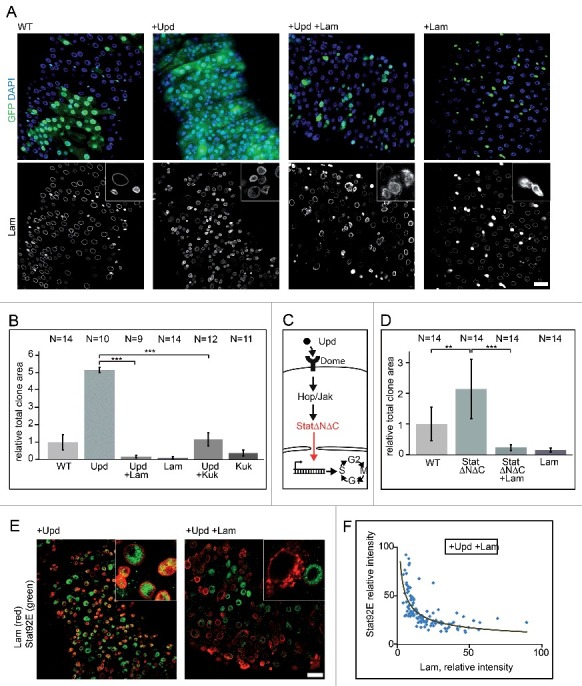
*Lam* and *kuk* overexpression inhibit *upd* induced proliferation. (A) Fixed guts with clonal expression of *unpaired* (*upd*) and *Lam* (+Lam) as indicated. Flipout clones (green, GFP), DNA (blue, DAPI), Lam (grey). Inset, 4x magnification. Five days of induction. (B, D) Quantification of clone size expressing GFP (WT), *Lam, upd* or *kuk*. The area with GFP expression marking the clone(s) in relation to total gut area within the field of view. Five days after clone induction. Bars represent standard deviation. N, number of guts. Statistical significance was tested by students T-test, two tailed, two-sample unequal variance. P(*upd* vs. *upd* +*Lam*) = 7.4×10^−20^, P(*upd* vs. *upd* +*kuk*) = 9.5×10^−16^, P(WT vs. StatΔNΔC) = 1,9×10^−3^, P(StatΔNΔC vs StatΔNΔC +*Lam*) = 1,2×10^−5^ (C) Scheme of the Jak/Stat pathway with constitutive induction at the level of Stat by expression of StatΔNΔC. (E, F) Midguts with flipout clones expressing *upd* or *upd*+*Lam* were fixed and stained for Stat (green) and Lam (red). Inset, 4x magnification. Five days of induction. (F) Fluorescence intensities of Stat and Lam staining in clonal cells plotted against each other. Linear regression yields a reciprocal correlation of y = 123.16x^−0.506^. Scale bars 25 μm.

The Jak/Stat pathway is one of several pathways that transduce proliferative signals in the midgut. Jak/Stat signaling is induced by the cytokine Unpaired (Upd) and its receptor Domeless (Dome) and plays an essential role in controlling ISC proliferation and tissue turnover under homeostatic and regenerative conditions. We induced ISC proliferation by Upd expression in flipout clones and assessed proliferation by clone size. Consistent with previous reports [21], this led to complete tissue turnover in less than five days. Coexpression of Upd and Lam led to a strong suppression of proliferation, indicating a suppression of Jak/Stat signaling (Fig. 6A, B and S5). A corresponding phenotype was observed with coexpression of Upd and Kuk (Fig. 6B and Fig. S6).

Lam may curb Jak/Stat signaling on multiple levels. It may have a direct effect on signaling by interfering with nuclear import of activated Stat, activation of Stat by Jak or impair the transcriptional activity of Stat. Alternatively, Lam may interfere indirectly by signal-independent effects on expression levels of Stat or Jak, for example. We tested whether Lam interferes with Jak/Stat signaling upstream or downstream of Stat in genetic terms. We expressed a constitutively activated form of Stat, StatΔNΔC [34], which does not depend on upstream activation for its downstream function (Fig. 6C) and scored the effect on proliferation by relative total clonal area. We found a moderate but significant activation of proliferation by StatΔNΔC, which was suppressed by Lam coexpression (Fig. 6D). These data suggest that Lam interferes with Jak/Stat signaling on the same functional level or downstream of Stat.

Stat is generally regulated by phosphorylation and nuclear import [35]. We first stained midgut tissue with a Stat antibody recognizing both phosphorylated and unphosphorylated forms. We detected nuclear localization of total Stat in ISC/EBs and overlapping staining with endogenous Lam even following Upd expression (Fig. 6E). However, after Lam overexpression, the strong Stat staining was lost. We detected an almost exclusive distribution of either Stat or Lam staining. Quantification of both staining intensities showed an inverse relation, with high Lam levels associated with low Stat levels and vice versa (Fig. 6F).

Secondly, we used an antibody specific for the phosphorylated form of Stat, pStat. Comparable to total Stat, pStat staining showed an inverse relation with Lam staining after Upd and Lam coexpression (Fig. S7). In control guts, Stat and pStat positive cells occurred inside and outside of GFP clones without prevalence. Upon expression of Upd, we detected strong Stat and pStat staining. Staining levels outside of the clones were mixed. The situation was different after coexpression of Upd and Lam. Stat and pStat staining was low inside of the clones whereas normal outside (Fig. S7). These data suggest that Lam affects total Stat protein levels, possibly by a transcriptional or post-transcriptional mechanism.

### Lamin overexpression normalizes Upd induced transcriptional response

**Figure 7.**
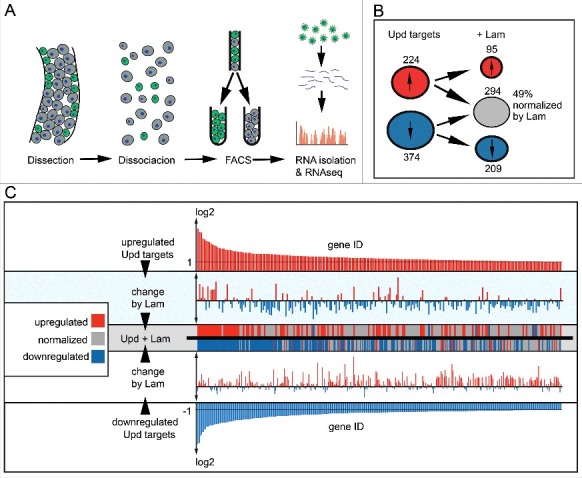
*Lam* overexpression normalizes expression of Jak/Stat target genes. (A) Experimental scheme for ISC/EB specific transcriptome analysis. Successful Lam overexpression in ISC/EBs was confirmed by significantly increased nuclear Lam immunofluorescence (Fig S8A). (B) Number of *upd* induced up- (red) and downregulated (blue) transcripts with (+*Lam*) or without *Lam* overexpression in ISC/EBs. (C) Change of *upd* target transcripts by *Lam* overexpression. Upper and lower panel show upregulated (red) and downregulated (blue) *upd* target genes sorted by changes in expression level. Middle panels show changes of each target gene upon *Lam* overexpression. Red indicates upregulation (higher than twofold), blue downregulation (lower then twofold) and gray return to normal expression (normalization).

Given its localization at the nuclear envelope, a likely activity of Lam is on the level of transcription. To test this option, we established transcriptional profiles of sorted ISC/EBs with and without Upd and Lam overexpression. Following isolation, midgut tissue with GFP labeled ISC/EBs cells was dissociated and sorted by FACS. Immediately after sorting, RNA was isolated and subjected to next-generation sequencing (Fig. 7A). We found that expression of Upd lead to an at least twofold upregulation of 224 transcripts and at least twofold downregulation of 374 transcripts (Fig. 7B, Fig. S8B). Overexpression of Lam lead to the downregulation of 1027 transcripts and upregulation of 673 transcripts (Fig. S8B). To identify a specific effect of Lam on the target genes of Upd/Jak/Stat signaling, we asked which Jak/Stat target genes would be changed by coexpression of Lam.

Surprisingly about half of the Jak/Stat target genes were normalized to levels within the two-fold threshold (Fig. 7C, genes marked by gray). Normalization in this context refers to the return of up and downregulated transcripts to their original wild type levels. Sorting of the Jak/Stat target genes according to the degree of up- or downregulation shows, that both strong and weak target genes were normalized by Lam overexpression (Fig. 7C). Only the very strong target genes as a class remained outside of the twofold threshold. In few cases, Lam even reverted upregulated transcripts of considerably high copy numbers to a level of significant downregulation. These data suggest that Lam has a distinctive, target specific, effect on the transcription profile of Jak/Stat target genes. It is therefore unlikely that Lam affects Stat levels by reduced nuclear import because this would result in an uniform normalization of all Jak/Stat targets.

Among the Jak/Stat target genes, whose expression was normalized by Lam coexpression was the Upd receptor Domeless (Dome). Dome is a Jak/Stat target with a 2.5-fold increased expression upon expression of Upd. Coexpression of Lam and Upd normalized Domeless expression to a factor of 1.49 (Fig. S8C). In summary, our transcriptional analysis indicates that Lam might act as a normalizing, rather than outright inhibiting factor on Jak/Stat signaling. The normalized levels of Dome may contribute to the anti-proliferative effect of Lam.

Lam may normalize the transcription response to Jak/Stat signaling by recruiting parts of the genome to the nuclear periphery. It has been reported previously that Lam interacts with specific heterochromatin-rich regions of the chromosome, designated Lamina associated domains (LADs) [36]. LADs are associated with gene silencing and potential regulation of developmental genes [37,38]. To test an involvement of LADs in normalization of Jak/Stat targets, we asked how many of the normalized target genes fall into LADs. Of the genes downregulated by Lam, 337 were found in LADs (35,7%), while 606 were found outside of LADs (Fig: S8D and S8E). The proportion of genes in LADs was comparable for upregulated genes (35.1%) and for all *Drosophila* genes (39.5%). Thus, we did not detect any specific link of the genes normalized by Lam overexpression and their association to LADs.

### *Lam* null clones do not show altered proliferation

The overexpression of *Lam* and *kuk* in ISC/EBs revealed an anti-proliferative activity. To test whether *Lam* and *kuk* also have a function in homeostasis and regeneration of the midgut tissue, we analyzed loss-of-function situations. *kuk*-deficient homozygous flies are viable and fertile [7]. We did not observe any differences to wild type flies in the number of ISC/EBs cells, indicating that *kuk* is not required for ISC proliferation. In contrast to *kuk, Lam* is partly required for viability [39] Only few *Lam*-null mutant flies reach the adult stage [40]. To test for a function of *Lam* in ISC proliferation, we induced *Lam* clones marked by GFP within a wild type gut. As *Lam* null clones were induced only in some ISCs, the clonal cells were in competition with wild type cells. Clonal cells contained no or only low levels of Lam persisting from the mother cell, as demonstrated by immunostaining (Fig. S9A). Clone size did not differ noticeably between wild type and *Lam* null clones even after 15 days. This indicates that Lam has no essential function in homeostasis of the midgut, what may be due to functional redundancy with other lamina proteins. Similarly, we could not detect a function of Lam in regeneration following bacterial infection. Firstly, we did not observe a difference in clonal area following *Pseudomonas entomophila* infection five weeks after clone induction (Fig. S9B). Secondly, proliferation was comparable in *Lam* null clones and wild type guts following infection with *Ecc15* bacteria for 10 h as we did not detect a difference in the mitotic index (Fig. S9C). These experiments indicate, that Lam is not required for proliferation of ISCs in the midgut during homeostasis and regeneration.

### Discussion

Expression of farnesylated lamina proteins can cause ageing-associated phenotypes and progeroid diseases in humans and animal models. For instance, expression of permanently farnesylated Lam A (Lam Δ50/Progerin) causes the Hutchinson Gilford Progeria syndrome (HGPS), which strongly reduces life span and causes several cellular and physiological effects reminiscent of ageing [1,3]How the cellular effects translate into physiological phenotypes is unclear. It has been hypothesized that impairment of stem cells function might play a significant role. Expression of Prelamin A or Progerin in mice leads to reduced stem cell numbers [10,11]. However, the link between farnesylated proteins stem cell proliferation is not well understood. In this study, we investigated the activity of the farnesylated lamina proteins Lam and Kugelkern in *Drosophila* flies. We have previously shown that overexpression of Lam or Kuk elicits ageing associated phenotypes on cellular and organismal levels, including shortened life span [8]. We now employed this experimental system to analyze the activity of Lam and Kuk in the well characterized stem cells of the midgut in adult flies. Lamina proteins show a stereotypic dynamic during differentiation of ISC/EBs. Kuk and Lam staining is strong in ISC/EBs and low or absent in differentiated EC. The function of the is dynamics is unclear. We found that overexpression of *Lam* and *kuk* in ISC/EBs and in clones strongly suppressed ISC proliferation. We detected a suppression of ISC proliferation under two conditions. During homeostasis, proliferation is low with a few mitotic cells per gut and an overall tissue turnover of about two weeks. In contrast, proliferation is high during regeneration, which we induced by bacterial infection. Despite this clear activity, *Lam* and *kuk* are not required for ISC proliferation. This might be due to functional redundancy with *LamC* or other lamina proteins. Comparable to mice mutant for the B-type Lamin [41], *Drosophila* flies homozygous for a null mutation in *Lam* are able to reach the adult stage [40].

The molecular mechanism by which *Lam* and *kuk* overexpression inhibits cell cycle progression is unclear. It unlikely that Lam and Kuk directly affect the basic cell cycle machinery, such as by activation of a checkpoint, depletion of cyclin, upregulation of a cell cycle inhibitor or remodeling of the cell cycle mode. We employed the FUCCI system, to score the distribution of the cell cycle stages in ISC/EBs but did not detect a change following *Lam* overexpression. The proportions of ISC/EBs in G1 and G2 phases remained largely unchanged. This finding suggests that Lam does not activate a checkpoint or affect a component of the cell cycle machinery. Alternatively, Lam may excert a more regulatory influence on the cell cycle. Supporting this notion is our finding that *Lam* overexpression did not suppress *Notch/Delta* induced proliferation. Activation of *Notch/Delta*, e g. by *Notch* RNAi, leads to a continuous, tumor-like proliferation of the ISCs. This type of proliferation was still observed when *Lam* was overexpressed. This finding indicates that the cell cycle can progress even when Lam is overexpressed. We propose a model where Lam acts on one or several specific signaling pathways that regulate cell cycle progression.

Multiple signals are involved in control of ISC proliferation [18,42]. Among these pathways, we focused on the Jak/Stat pathway, which is induced by the cytokine Unpaired. The Jak/Stat pathway is active under homeostatic and regenerative conditions. In contrast to Notch/Delta induced proliferation, cytokine induced proliferation was fully suppressed by *Lam* overexpression. Lam may suppress Jak/Stat signaling in two ways. Firstly, we observe a downregulation of Stat protein in cells overexpressing *Lam*. As we do not observe an effect of Lam on the expression of Stat RNA, the downregulation of Stat protein may be due to reduced translation or increased degradation of Stat. Secondly, Lam modulates the transcriptional response of Jak/Stat signaling. Half of the Jak/Stat target genes in isolated ISC/EBs were normalized by concomitant Lam overexpression. Interestingly, among the Jak/Stat target genes that were normalized by Lam is the cytokine receptor Domeless. This suggests that among other effects, the missing upregulation of the cytokine receptor leads to a weakened sensitivity of ISCs for cytokine signals. Furthermore, our data further suggests that some Jak/Stat targets are affected more than others. This finding excludes models which involved global effects on Jak/Stat signaling upstream of the transcriptional response, e. g. inhibition of nuclear import of Stat.

**Figure 8.**
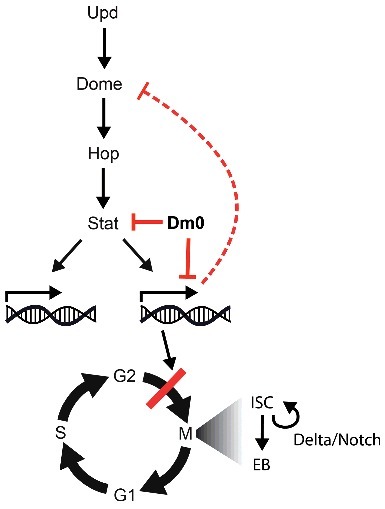
Model. *Lam* overexpression antagonizes Stat protein levels and normalizes Jak/Stat target gene expression, including normalization of *domeless* expression. As a consequence of reduced Jak/Stat signaling, and possibly other pathways, Notch/Delta independent proliferation regulation is suppressed.

We propose a model in that Lam overexpression inhibits ISC proliferation without depleting them. The effect of Lam overexpression is mediated in two ways. Lam reduces Stat protein levels posttranslationally and normalizes the expression of about half of the Jak/Stat target genes. Among the affected Jak/Stat targets is the Upd receptor Domeless. Reduction of Domeless leads to increased insensitivity of ISCs to Upd which further reduces proliferation (Fig. 8).

## Materials and methods

### Genetics

Fly stocks were obtained from the *Drosophila* stock center in Bloomington, if not otherwise noted (Table 1). Genetic markers are described in Flybase [43] if not otherwise noted. OregonR was used as wild type. If not otherwise noted, adult female flies were analyzed 2–6 d after eclosion. Expression specific in ISC/EB cells was achieved with the Esg-GAL4TS driver line, which was crossed to a UASt transgene. Expression was controlled by temperature shift from room temperature to 29°C. The flies contained a temperature sensitive version of GAL80, GAL80TS, which binds and suppresses GAL4. At high temperature (29°C) GAL80TS is instable and thus allows activation of gene expression by GAL4 and corresponding activation of UAS target genes. Expression in ISC/EB and clones was achieved with a flip-out cassette (EsgTS;FO). Flipase expression induces mitotic recombination (flipout) of a stuffer sequence. This allows tubulin-GAL4 expression and corresponding activation of UAS target genes. Flipout clones were induced by permanent shift to 29°C for five days, if not otherwise noted. MARCM clones (positively marked loss of function clones) were induced by mitotic recombination of centromeric FRT site on the left arm of the second chromosome. Mitotic recombination was activated by heatshock activated expression of a flippase (shift to 37°C for one hour on three consecutive days).

### Cloning

Full length *kuk* cDNA was transferred as a NotI-EcoRI fragment from the plasmid pMT-Kuk to the pUASt. Transgenic flies were generated by standard P element mediated insertion into the genome. Insertions on the second or third chromosome were isolated.

### Histology

Following dissection from adult flies, complete guts were fixed in 0.2% Tween-20, 0.5% Nonidet P40, 8% formaldehyde, in phosphate buffered saline (PBS) for 40 min. After rinsing in PBS-T (PBS with 0,1% Tween), the fixed gut tissue was permeabilized in PBS with 0.5% Saponin and 0.5% Triton-X100 overnight. After rinsing with PBS-T and blocking with 5% bovine serum albumin in PBS-T for 40 min, the primary antibody (Table 2) diluted in PBS-T was applied at room temperature for two hours or at 4°C overnight. Following 3x rinsing with PBS-T and washing in PBS-T for 1 h, the secondary antibody was applied at room temperature for 2 h or at 4°C overnight. After rinsing 3x in PBS-T and washing in PBS-T for 1 h, the stained gut tissue was mounted on a glass slide in 47.5% glycerol, 47.5% PBS, 5% 1,4-diazabicyclo[2.2.2]octane (DABCO), covered with a cover slip and sealed with nail polish. Secondary antibodies were labelled with Alexa dyes and applied at a dilution of 1:500 (Invitrogene). F-actin was stained with Alexa-linked phalloidin (Invitrogene).

### Microscopy

About ten guts per genotype were imaged in parallel with the tile scan option with a EC Plan-Neofluar 10x/NA 0.3 objective on a laser scanning confocal microscope (LSM780, Zeiss). Images at high resolution were recorded images with a LCI Plan-Neofluar 63x/NA1.3 objective with water/glycerol immersion.

### Western blot

Expression in the mid gut was induced in enterocytes with the driver line myo1B-GALTS. After five days of induction, guts were dissected and immediately frozen and stored in liquid litrogen. The frozen guts were covered with 4x Laemmli buffer and incubated at 95°C for 10 min. For mechanical dispersion, the extracts were pipetted 30 times up and down, dissolving gut tissue and sheering genomic DNA. Extracts were subsequently incubated at 95°C for 5 min and briefly centrifuged. An equivalent of three guts was analyzed per lane by SDS polyacrylamide gelelectrophoresis. Loading was controlled the ubiquitously expressed enzyme serine hydroxymethyl transferase (SHMT), which is constitutively expressed in embryos [52].

Following antibodies were employed, Kuk (rabbit, 1:12500, [7]), SHMT (guinea pig, 1:7500 [52]), Lam (mouse, 1:750), LamC (mouse, 1:500). Western blots were developed with fluorescently labelled secondary antibodies (1:20000) and optical detection (Licor). 16 bit images were processed with Photoshop.

### Quantification of clones size

Relative clone size: Fiji [53] Threshold function was used to define GFP positive clones, then the whole midgut area was encircled with the lasso tool and the mean fluorescence measured. Measurements of all genotypes were displayed relative to the average clonal area of the control flies. Statistical values of respective genotypes were divided by WT values (WT represented by 1) to represent ratios of increase/decrease. Error bars signify standard deviation. Statistical significance was tested by students T-test, two tailed, two-sample unequal variance.

### Analysis of gene expression in ISC and enteroblasts

Four genotypes (1. EsgTS/CyO; Dr/TM3, 2. EsgTS; UAS-Lam/+ 3. EsgTS/Upd 4. EsgTS/Upd; UAS-Lam/+ were analyzed with three biological replicates. For each genotype, 3 × 100–150 guts were dissected. For dissection the last segments of the abdomen were pulled apart by micro-forceps, exposing the posterior midgut and hindgut. The front segments of the abdomen were pulled apart from the thorax to expose most of the anterior part of the midgut. The remaining anterior part of the midgut, together with foregut and crop, were pulled carefully out of the thorax. The crop and foregut was removed and the gut pulled through the anterior part of the abdomen. The malpighian tubules, hindgut, rectum and the posterior segments of the abdomen were removed and the midgut transferred to a 1.5 ml reaction vial, on ice, containing 400 *µ*l of PBS. Cells were dissociated by adding 100 *µ*l of Elastase solution to a final concentration of 0.8 mg/ml and incubation at 27°C for 1 h with gentle agitation every 15 min and 40 times pipetting up and down. After centrifugation at 600xg at 4°C for 10 min, the cells were suspended in 500 *µ*l PBS. Intestinal cells and enteroblasts were sorted by flow cytometry according to cell size and GFP fluorescence in the Scientific Flow Cytometry Facility. The escGAL driver induces GFP expression specifically in intestinal stem cells and enteroblasts. The dissociated gut tissue was applied to a 50 *µ*m cell-sieve, removing the large enterocytes and cellular debris. The remaining cells were sorted for granularity, excluding damaged or clumped cells, and GFP fluorescence. The typical yield was 600 ISC/EBs per gut. The cells were sorted directly into RNA extraction buffer from the PicoPure RNA isolation kit, with which RNA was isolated. Following incubation at 42°C for 1 h, 500 *µ*l 70% ethanol was added. The mixed solution was split in two samples and applied to two RNA extraction columns. The columns were centrifuged at 100×g for 2 min, and at 16000×g for 30 s. The flow-through was discarded. Columns were washed with 100 *µ*l Wash buffer 1 and centrifuged at 8000×g for 1 min. RNA was eluted with elution buffer. Samples of the two corresponding columns were pooled and frozen. Total polyA RNA was selected and subjected to library preparation for Illumina ”sequencing-by-synthesis” technology. Next generation sequencing and bioinformatics analysis was conducted by the transcriptome core laboratory. Clustering of RNAseq data was controlled by principle component analysis.

### Bacterial infection

100 ml LB-medium was inoculated with *Erwinia carotovora carotovora* (*Ecc15*), *Pseudomonas entomophila* (*P. e*.) or *E. coli* (DH5α) bacteria and incubated at 30°C, overnight. Cells were isolated by centrifugation (3466×g, 15 min), suspended in 5 ml PBS with 5% saccharose and mixed with the upper food layer of a small fly food vial (lacking the preservatives, propionic acid). To avoid flies sticking to the food, a piece of filter paper was placed into the vial without making contact to the food. Flies were starved without food and water for 6–10 h prior to transfer to the infection vial. After 12 h, flies were dissected in the infection vial. *P.e*. bacteria were tested for pathogenicity by spreading a diluted pellet sample on a agar plate with 1% skimmed milk powder. The plate was incubated for 24 h at 30°C. Pathogenic *P.e* colonies with proteolytic activity were distinguished by a clear area around the colony.

**Table 1.** *Drosophila* stocks used in this work.

Name	Genotype	Source	Reference
EsgTS	Esg-Gal4, tub-GAL80TS, UASt-nlsGFP	B. Edgar	[15]
EsgTS; FO	Esg-Gal4, tub-Gal80TS, UASt-nlsGFP; act>CD2>Gal4, UASt-flp	B. Edgar	[21]
Upd	UASt-Upd26.2	B. Edgar	[21]
Lam	UASt-Lamin	M. Krasnow	[44]
Upd; Lam	UASt-Upd26.2; UASt-Lamin	this work	
LamC-CaaX	UASt-LamC-CaaX	G. Krohne	[45]
MARCM	hsFLP,UASt-CD8-GFP; tub-Gal80FRT2L; tub-Gal4	Bloomington	FBst0042725
FRT2L	FRT2L	Bloomington	FBti0002071
LamD395	FRT2L::LamD395	Y. Zheng	[39, 46]
FUCCI	UASp-GFP-E2F1, UAS-mRFP1-NLS-CycB	Bloomington	[33] FBst0055100
FUCCI; Lam	UASp-GFP-E2F1, UAS-mRFP1-NLS-CycB; UASt-Lam	this work	
Kuk	UASt-Kugelkern	this work	
StatΔNΔC	UASt-Stat92EΔNΔC	E. Bach	[34]
StatΔNΔC; Lam	UASt-Stat92EΔNΔC; UASt-Lam	this work	
Notch RNAi	UASt-Notch RNAi	Vienna RNAi Centre	FBst0471876
MyoIA-GAL4	MyoIA-Gal4; tub-GAL80TS, UASt-nlsGFP	B. Edgar	[21]

**Table 2.** Primary antibodies.

Antibody	Dilution	Source	Ref.
mouse-α-Lam	1:250	H. Saumweber	[47]
mouse-α-LamC	1:250	Hybridoma Bank	AB 528339
rabbit-α-Kuk	1:1000	J.Großhans	[7]
guinea-pig-α-Lam C-terminus	1:250	G. Krohne	Raised against 415–622 aa residues
rabbit-α-Stat92E	1:1000	SX. Hou	[48]
mouse-α-pStat92E	1:2000	X. Lin	[49]
mouse-α-PH3	1:1000	Millipore-Upstate	Cat. # 05–806
guinea-pig-α-Otefin	1:750	G. Krohne	[50]
rat-α-Klaroid	1:750	M. Welte	[51]
rabbit-α-Nup50	1:10000	J. Großhans	[7]
mouse-α-Delta	1:25	Hybridoma Bank	AB 528194

## Supplementary Material

2017NUCLEUS0052R-s02.docx

## Data Availability

RNA seq data: GEO Submission number (GSE97307) [NCBI tracking system #18363487].

## Summary statement

Expression of Lamin or Kugelkern curb stem cell proliferation by suppression of Jak/Stat signaling.
