# Supplementary material for: Expression of lamina proteins Lamin and Kugelkern suppresses stem cell proliferation
Source: Nucleus. 2018 Jan 23;9(1):104–18. doi: 10.1080/19491034.2017.1412028 (PMC5973253; doi:10.1080/19491034.2017.1412028)
Supplement: 2017NUCLEUS0052R-s02.docx [file kncl-09-01-1412028-s001.docx]

**Supplemental materials**

**Expression of lamina proteins Lamin and Kugelkern suppresses stem cell proliferation**

Roman Petrovsky and Jörg Großhans


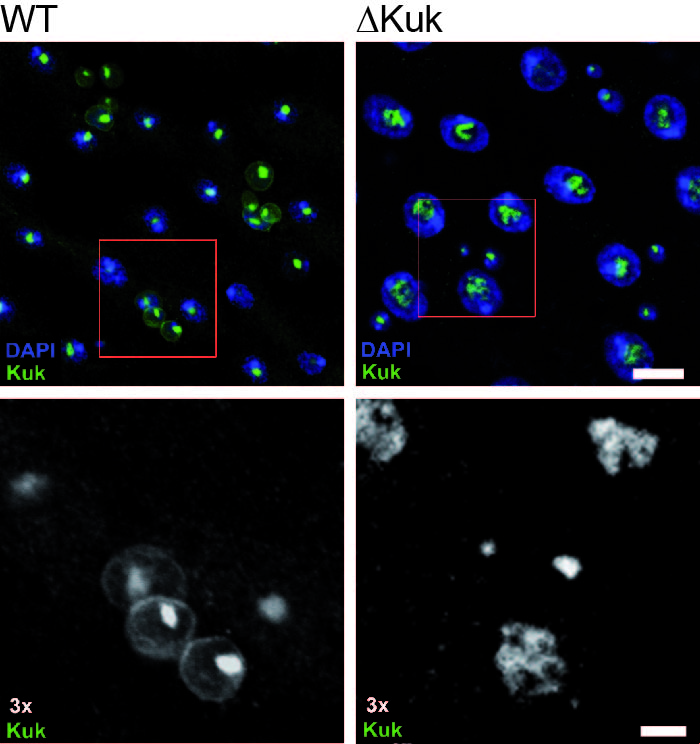


**Figure S1: Localization of Kuk in WT and Kuk deficient flies.**

Midguts OrR (WT) and Kuk∆15 (∆Kuk) stained for DNA (blue), Kuk (green, grey as indicated) Red square indicates 3 x enlarged section (lower panel). Scale bar: 10 *μ*m upper panel, 3 *μ*m lower panel. (Images from Dissertation, Roman Petrovsky “Role of the nuclear lamina for stem cell mediated homeostasis”)


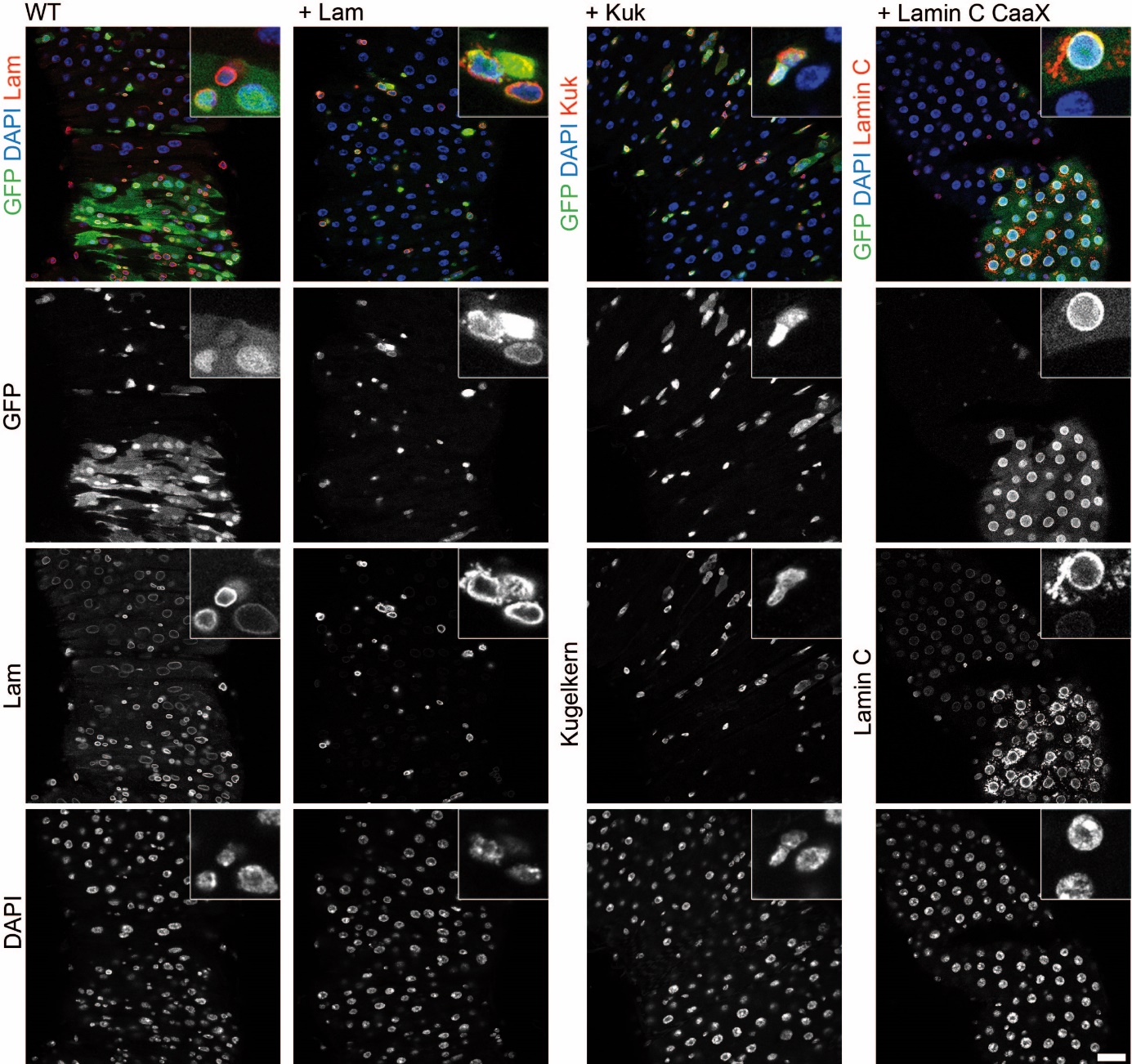


**Figure S2: *Lam* overexpression inhibits ISC proliferation.** Midguts with clonal expression of GFP or GFP and *Lam*, *Kuk* or *LamC-Caax* as indicated and stained for DNA (blue), GFP (green) and Lam, Kuk and LamC (red). Five days of clone induction. Insets, 4x magnification­­. Scale bar: 25 *μ*m

**:**


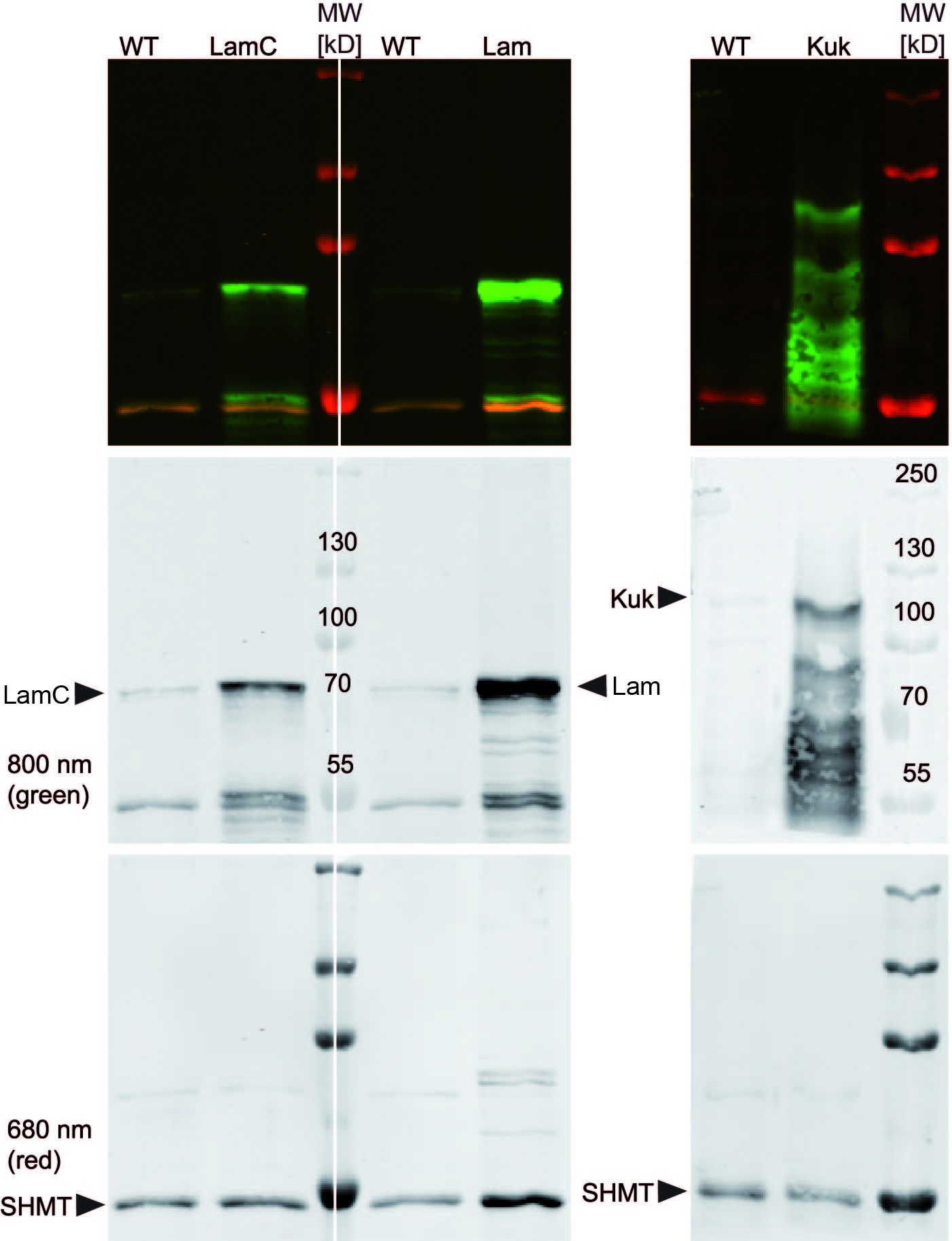


**Figure S3: Western blots of Lam, LamC and Kuk overexpression.**

LamC-CaaX, Lam and Kuk overexpressed for five days in ECs (MyoTS driver). Expression levels were analysed by western blots with total gut extracts. Kuk overexpression resulted in strong fragmentation possibly due to the fact that Kuk does not localize at the nuclear lamina in ECs. SHMT serves as loading control.


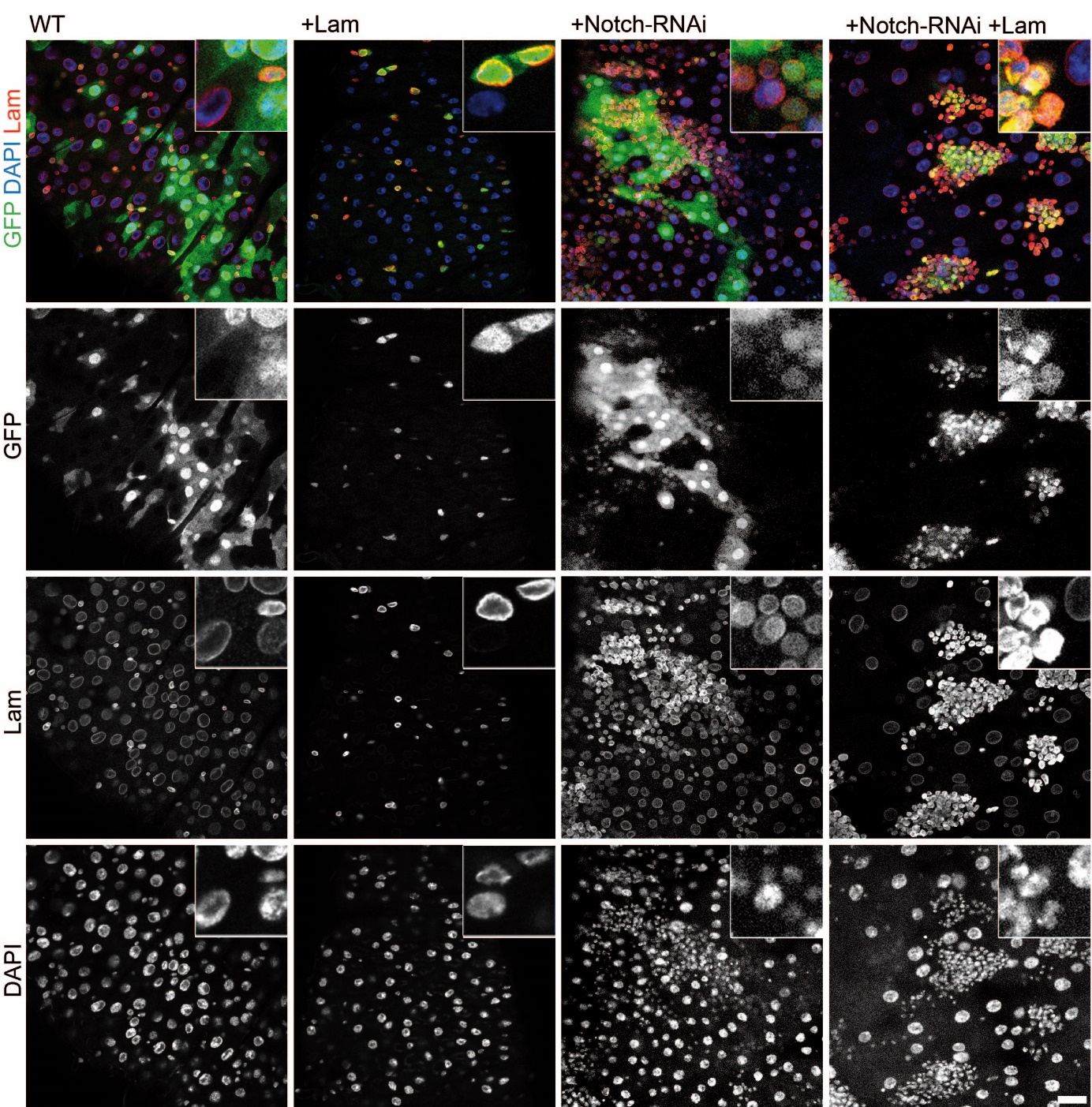


**Figure S4: *Lam* overexpression does not suppress *Notch/Delta* induced proliferation.** Midguts with clonal expression of GFP, *Lam* and *Notch* RNAi as indicated and stained for DNA (blue), Lam (red), GFP (green). Five days of clone induction. Insets, 4x magnification. Scale bar: 25 *μ*m


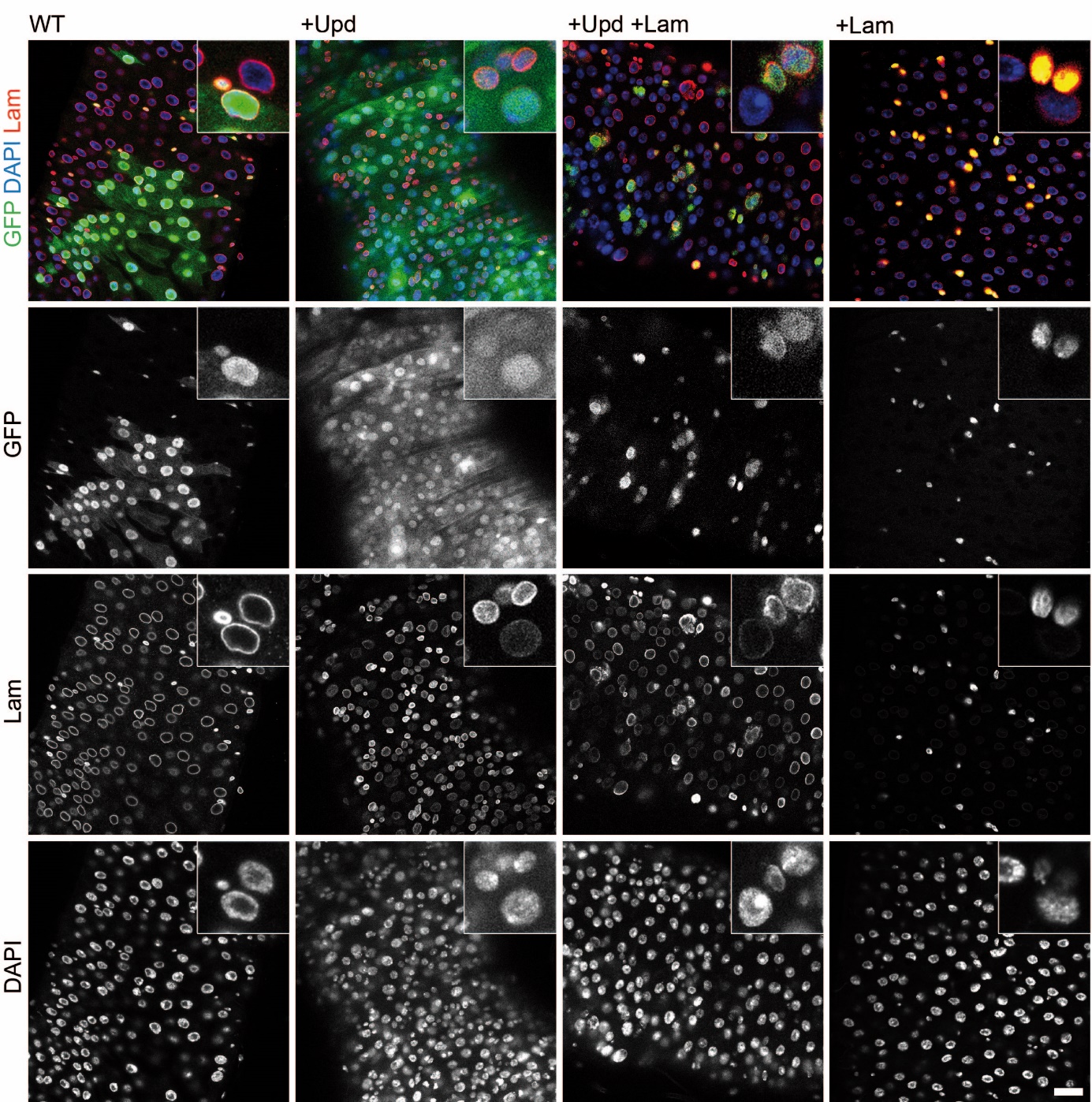


**Figure S5: *Lam* overexpression inhibits *Upd* induced proliferation.** Midguts with clonal expression of GFP, *Unpaired (Upd*) and *Lam* as indicated stained for GFP (green), DNA (blue), Lam (red). Single channels as indicated in greyscale. Insets, 4x magnification. Five days of clone induction. Scale bar: 25 *μ*m


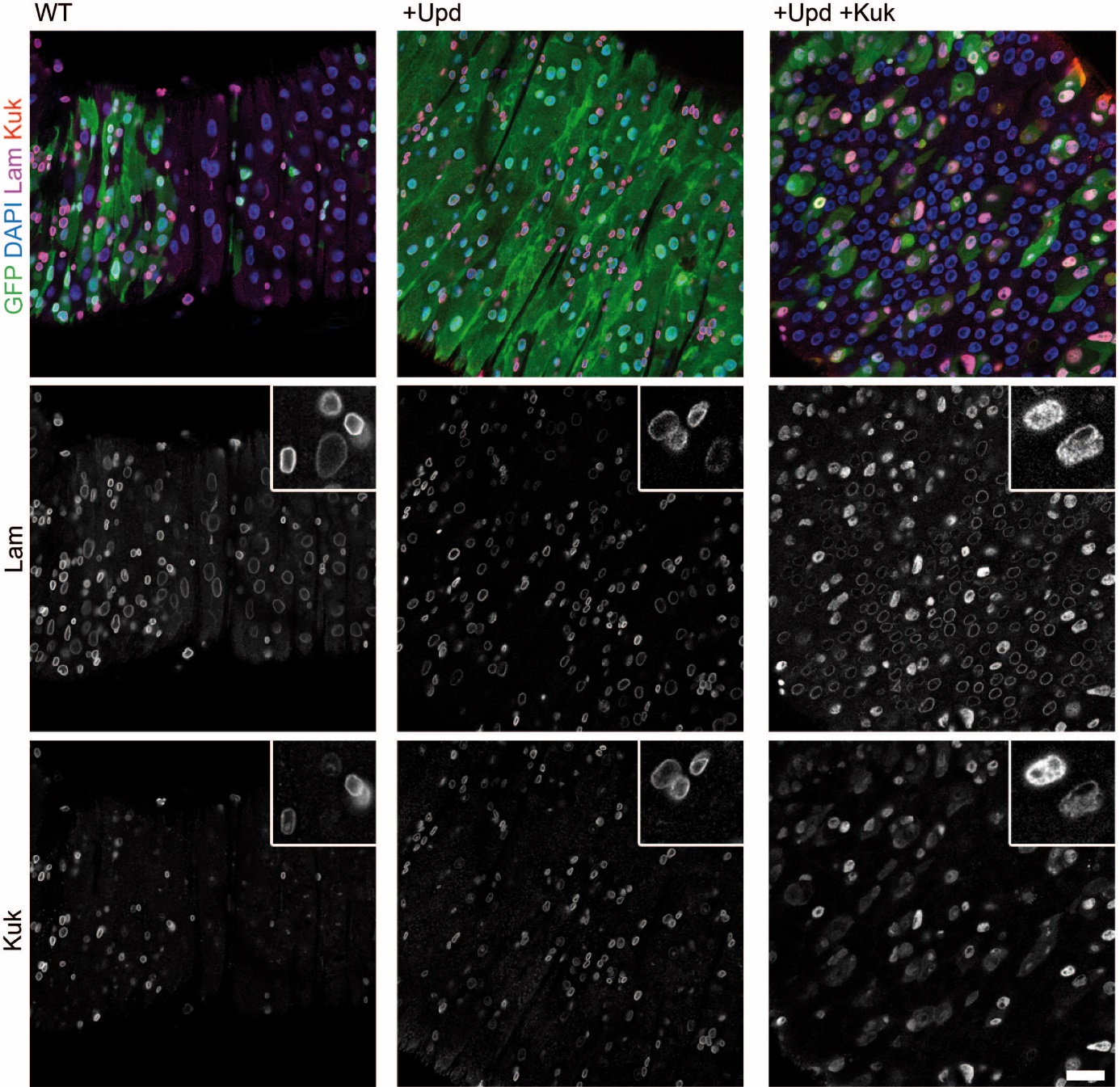


**Figure S6: *Kuk* overexpression curbs Jak/Stat induced proliferation.** ­­Midguts with clonal expression of GFP (WT), *Unpaired* (+*Upd*) and *Kuk* as indicated and stained for GFP (green), DNA (blue), Lam (purple, gray) and Kuk (gray). Single channels as indicated in grey scale. Five days of clone induction. Insets, 3x magnification. Scale bar: 25 *μ*m


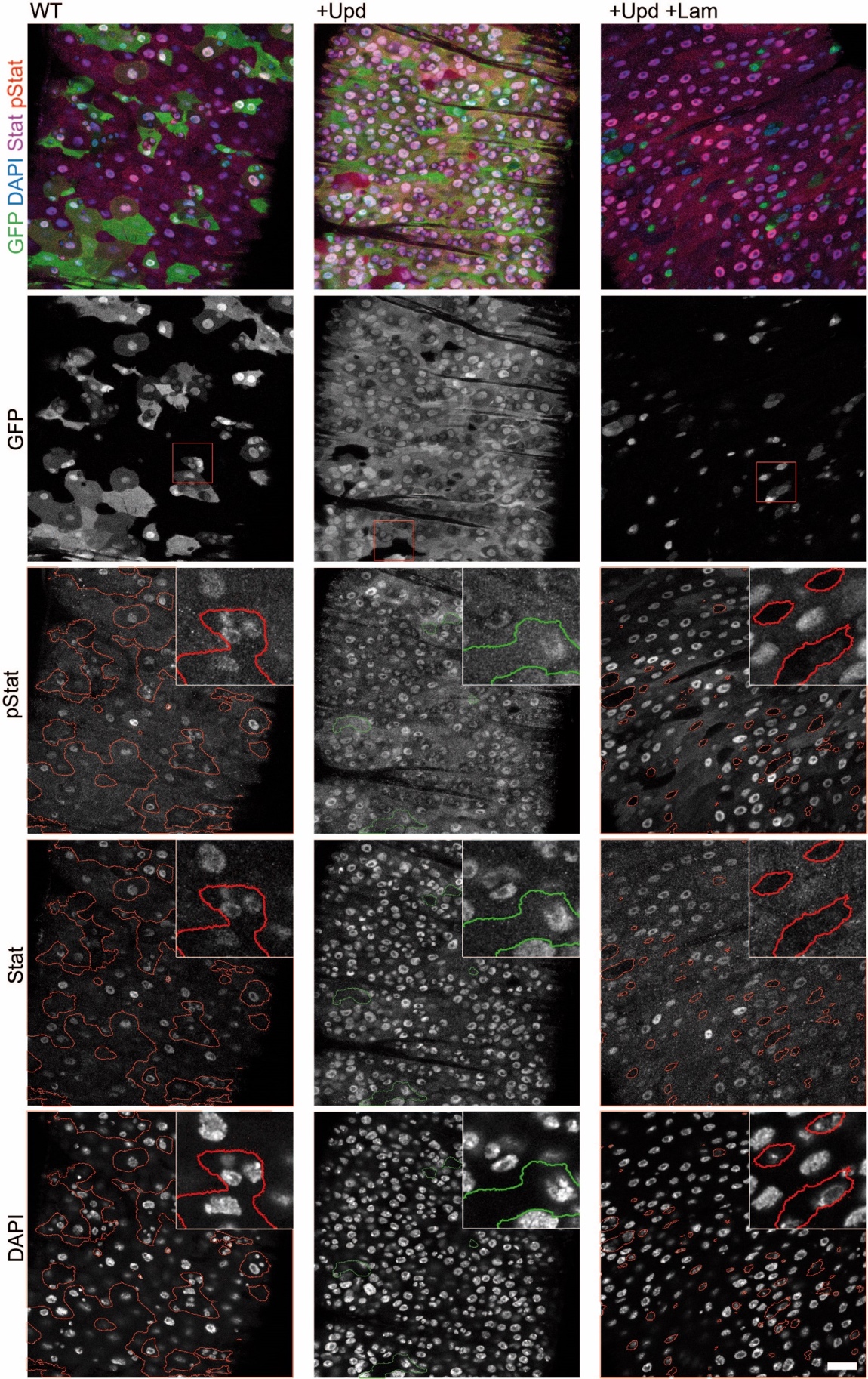


**Figure S7: *Lam* overexpression antagonizes Stat and pStat protein levels.** Midguts with clonal expression of GFP and *Upd* or *Lam* as indicated and stained for GFP (green), DNA (blue) phospho-Stat (red) and Stat (purple). Single channels as indicated in greyscale. Clonal area is marked by line in red. The line in green marks the non-clonal area in guts with clonal *Upd* expression. Five days of clone induction. Insets, 3x magnification. Scale bar: 25 *μ*m


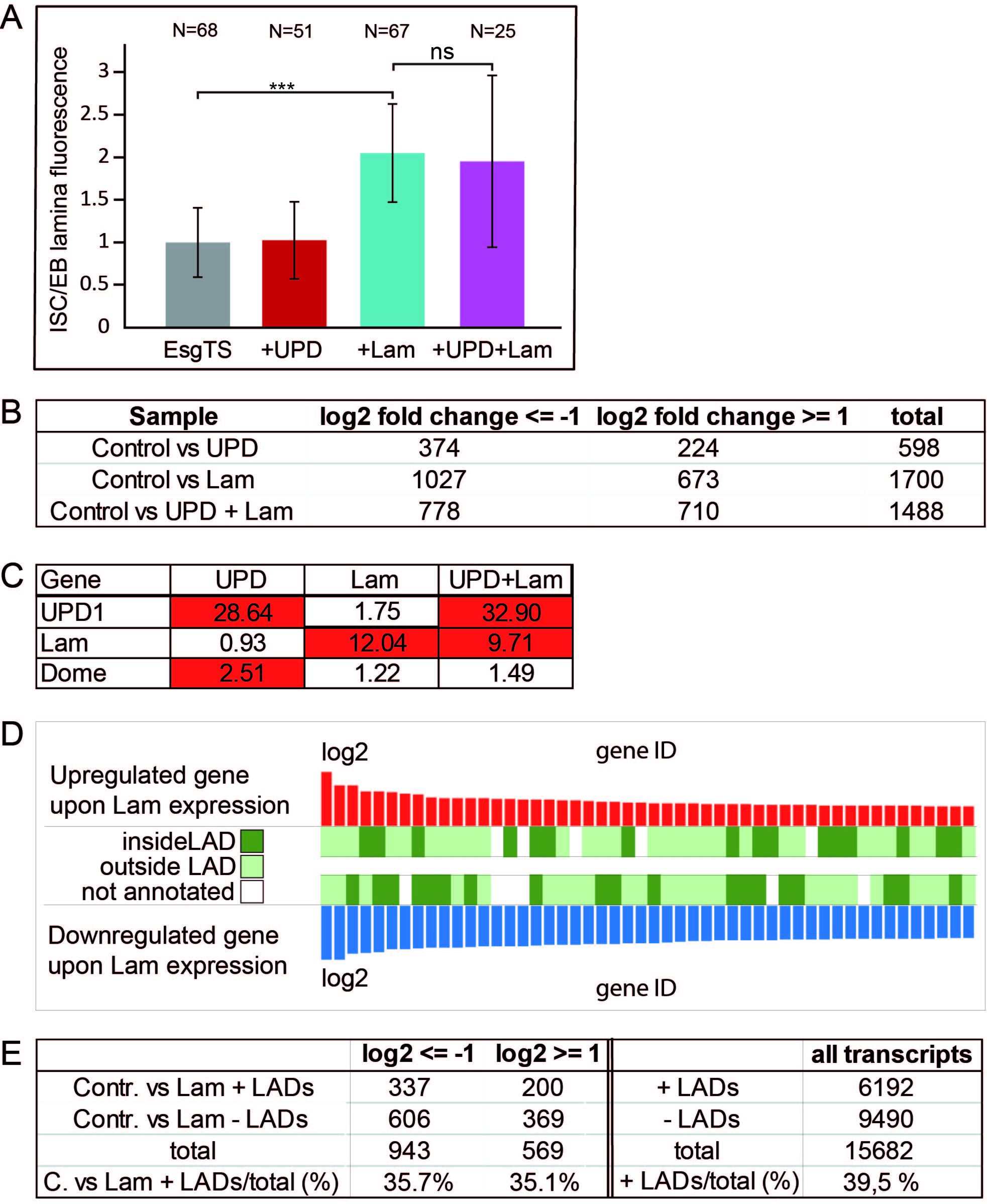


**Figure S8: Transcriptional profiling.** Results of RNAseq experiment of EsgTS (control) flies with expression of *Upd, Lam* or *Upd* and *Lam* in ISC/EBs. A: Quantification of Lam immunofluorescence intensity in ISC/EB nuclei. For each image the average nuclear immunofluorescence intensity of Lam was determined for ECs and subtracted from each ISC/EB cell (marked by GFP). N= Number of ISC/EBs measured. Statistical significance was tested by students T-test, two tailed, two-sample unequal variance. P(WT vs. +Lam) = 6.4*10-23, P(+Lam vs. +UPD+Lam) = 0.65. B: Number of transcripts that were downregulated or upregulated upon expression of *Upd, Lam* or both, in ISC/EBs with GFP expression, compared to only GFP expressing ISC/EBs. C: Selected genes that are upregulated (red) or unchanged/normalized (white). Numbers describe fold change in expression (not log2) D: Upregulated (red) or downregulated (blue) transcripts in relation to LADs (inside, outside or not annotated). Shown are the top 50 up/downregulated transcripts upon Lam expression. E: Left part of the table shows the number of transcripts that are downregulated (log2 <=-1) or upregulated (log2 >=1) upon *Lam* expression and if the gene locus is inside a LAD (Contr. vs *Lam* + LADs) or outside a LAD (Contr. vs *Lam* - LADs). The right part of the table shows all annotated *Drosophila* genes that lie in LADs (+LADs) and outside of LADs (-LADs) and the percentage of those that lie in LADs (+ LADs/_total_ (%)).


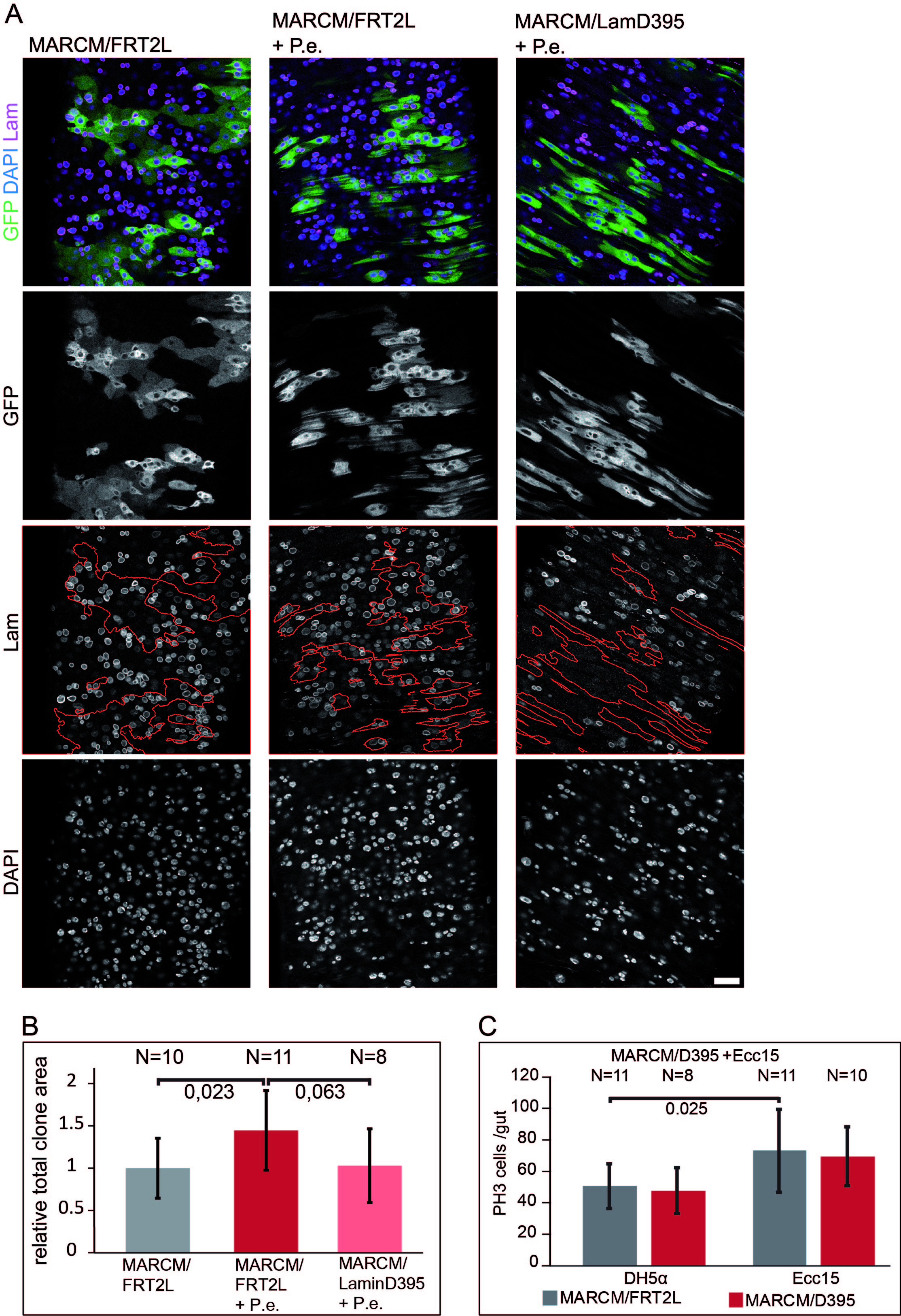


**Figure S9: *Lam* is not required for proliferation in midgut.** Midguts with mitotic clones for wild type or a *Lam* mutation were infected with *Pseudomonas entomophila (P. e*.) as indicated and (**A, B**) stained for GFP (green clonal marker, DNA (blue), Lam (purple). Clones are marked by line in red. After five weeks of clonal growth flies were infected by *P.e.* for 5 d and by *Ecc15* for 12 h and fixed afterwards. WT= MARCM/FTR2L, ∆Lam=MARCM/*Lam*^D395^ **(B)** Quantification of clonal area per gut region. P(WT vs. WT + *P. e.)* = 0.023, P(WT + P. e. vs. Lam + *P.e.*) = 0.063.  **(C)** Mitotic index as determined by staining with the mitotic marker (pH3). Flies with wild type or *Lam* mutant clones were infected with non-pathogenic (DH5a) or pathogenic (*Ecc15)* bacteria after four weeks of clonal growth. Bars indicate standard deviation. N, number of guts. Statistical significance was tested with Students T-test, two tailed, two-sample unequal variance. P(WT + DH5a vs. WT + *Ecc15*) = 0.025. Scale bar: 25 *μ*m.
